# BioMoby extensions to the Taverna workflow management and enactment software

**DOI:** 10.1186/1471-2105-7-523

**Published:** 2006-11-30

**Authors:** Edward Kawas, Martin Senger, Mark D Wilkinson

**Affiliations:** 1 James Hogg iCAPTURE Centre for Cardiovascular and Pulmonary Research, St. Paul's Hospital, Department of Medical Genetics, University of British Columbia, #166 – 1081 Burrard St., Vancouver, BC, V6Z 1Y6, Canada; 2 International Rice Research Institute (IRRI), Bioinformatics Unit, Manila, Philippines

## Abstract

**Background:**

As biology becomes an increasingly computational science, it is critical that we develop software tools that support not only bioinformaticians, but also bench biologists in their exploration of the vast and complex data-sets that continue to build from international genomic, proteomic, and systems-biology projects. The BioMoby interoperability system was created with the goal of facilitating the movement of data from one Web-based resource to another to fulfill the requirements of non-expert bioinformaticians. In parallel with the development of BioMoby, the European myGrid project was designing Taverna, a bioinformatics workflow design and enactment tool. Here we describe the marriage of these two projects in the form of a Taverna plug-in that provides access to many of BioMoby's features through the Taverna interface.

**Results:**

The exposed BioMoby functionality aids in the design of "sensible" BioMoby workflows, aids in pipelining BioMoby and non-BioMoby-based resources, and ensures that end-users need only a minimal understanding of both BioMoby, and the Taverna interface itself. Users are guided through the construction of syntactically and semantically correct workflows through plug-in calls to the Moby Central registry. Moby Central provides a menu of only those BioMoby services capable of operating on the data-type(s) that exist at any given position in the workflow. Moreover, the plug-in automatically and correctly connects a selected service into the workflow such that users are not required to understand the nature of the inputs or outputs for any service, leaving them to focus on the biological meaning of the workflow they are constructing, rather than the technical details of how the services will interoperate.

**Conclusion:**

With the availability of the BioMoby plug-in to Taverna, we believe that BioMoby-based Web Services are now significantly more useful and accessible to bench scientists than are more traditional Web Services.

## Background

The BioMoby project [1-3] began in 2001 as an initiative of the model organism database community and other interested parties to define standards and technologies that would facilitate greater interoperability between Web-based bioinformatics and biological resources. It now boasts greater than 50 independent host providers spanning five continents, and offering more than 800 data retrieval and analysis services.

The interoperable behaviors observed between BioMoby services are derived by the service-providers adherence to an ontology-based messaging structure that allows both BioMoby clients and BioMoby services to look-up data-types in the ontology, and thereby interpret and parse them correctly. In addition to this, an ontology-aware registry, MOBY Central, is capable of brokering the interactions between a client holding a particular data-type and/or with specific analytical requirements and the service providers who can operate over that type of data, or ontologically compatible types, through queries over the relevant ontologies. In this way, MOBY Central can suggest a subset of Services from its database which are guaranteed to be capable of consuming the client's in-hand data.

BioMoby-compliant Web Services are becoming increasingly easy to design and deploy thanks to a myriad of tools available to service providers through the open-source BioMoby project, and collaborating projects such as myGrid [4,5] and the Generation Challenge Programme of the Consultative Group for International Agricultural Research [6]; however, until recently, the full capabilities of the BioMoby system have not been available through any publicly available client program, and thus many of the most useful behaviors required custom programming to access.

Numerous clients have been created to utilize BioMoby (Gbrowse Moby [13], MOWServ [14], Remora [15], BlueJay [16], SeaHawk [17]). Most of these clients utilize the BioMoby Application Programming Interface (API) in a Web interface, allowing users to start with a piece of data, a GenBank gene identifier for example, and then discover services that consume that piece of information, followed iteratively by discovery of services that consume the output from the previous service, and so on. Some clients allow you to create and save workflows of services that can be re-used at a later date. So far, however, none of these clients have been capable of enabling interoperability between BioMoby services and non-BioMoby services, or of assembling and/or decomposing BioMoby data types at any point in the workflow to enhance service discovery.

The Taverna [7-9] workflow and enactment system from the myGrid project can "converse with the interfaces of Web Services and direct the flow of data between resources" [7]. While Taverna was capable of accessing most typical Web Services, allowing end-users to drag-n-drop resources into a pipeline, it had not been able to exploit the BioMoby ontologies to enhance the process of service discovery. Importantly, Taverna was not able to verify if services added to the workflow were truly capable of consuming the output data from the previous service, nor could it (alone) suggest which services might be capable of doing so. As the number of resources available through the Taverna interface continues to increase (currently more than 6000!) this lack of guidance through the vast Service-space has been a high barrier to entry for the naïve end-user.

**Figure 1:**
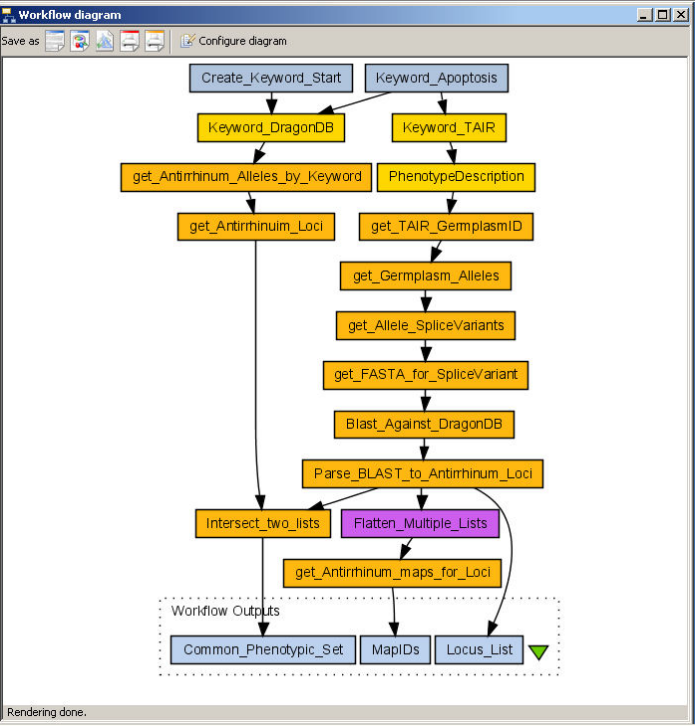
A BioMoby workflow. The function of this workflow is to retrieve the list of loci from *Antirrhinum *(Snapdragon) and *Arabidopsis *which share both a keyword in their phenotypic description as well as sequence homology of the affected locus, thus more likely representing true homologues. Yellow boxes represent BioMoby Objects; orange boxes represent BioMoby services; white boxes represent BioMoby Object parsers; Purple boxes represent local Java Bean widgets; Blue boxes represent inputs and outputs.

With this in mind, we developed extensions to Taverna that allow BioMoby to guide the construction of functional workflows. Moreover, the logic for decomposition of BioMoby data-types into their component parts, and/or the aggregation of data from multiple workflow branches (BioMoby-based or otherwise) into complex BioMoby data-types, and the subsequent utilization of these in the workflow is also enabled. Finally, a generic BioMoby data parser has been included that allows raw data to be extracted from BioMoby-XML and passed into traditional Web Service interfaces. For example, the workflow diagrammed in Figure 1 was constructed using the BioMoby plugin and shows the initiation of the workflow with a complex data-type, the pipelining of that data through a series of BioMoby Services, followed by the parsing of that data into a non-BioMoby service, with the outputs gathered into various output data bins. Here we describe these functionalities in detail, and argue that, with these functionalities present in the most widely used bioinformatics Web Service client, BioMoby services are now significantly more useful than traditional Web Services.

## Implementation

The 1.4 and greater releases of Taverna include the BioMoby plug-in and these are implemented as a Java 1.5 standalone application. The BioMoby plug-in implements the IProcessor interface of the scufl package to describe BioMoby services in Taverna. In order to run BioMoby services in Taverna, all services must also implement the ProcessorTaskWorker interface described in the scuflworkers package. Finally, in order for the workbench to discover BioMoby services, the BioMoby plug-in extended the Scavenger class from the scuflui package.

## Results

### Traditional Web Services versus BioMoby Services

The key difference between traditional Web Services and BioMoby services is in the definitions of the input and output data structures. Traditional Web Services utilize XML schema to describe the basic syntax of their interface [10], but not its "intent". As such, an interface might define, for example, an xsd:String as one of its input parameters; however there is no way for a client program to determine if that String is intended to be a DNA Sequence or a PubMed abstract or any of the thousands of other bioinformatics data-types that are commonly represented as strings. To overcome this problem, BioMoby defines an ontology of bioinformatics data-types [11], and a XML representation of this ontology [12] such that the position of a node in the ontology precisely defines the XML syntax by which that node will be represented, and the name and definition of the node define precisely the purpose of that syntax – i.e. the nature of the data it will contain. Thus it is possible for a client program to precisely query the MOBY Central registry for services that operate with the in-hand data-type, and moreover, that in-hand data can be passed *verbatim *to the service provider, thus not requiring the client to include any complex data-rearrangement code.

### The core Taverna interface

The Taverna interface includes three main windows:

• The Available Services window provides a menu of possible widgets to add to a workflow, including both local Java Bean widgets, as well as remote Web Services. These are hierarchically organized based on the Web Service registry or WSDL file that contains their definitions.

• The Advanced Model Explorer window provides details of all services and service-to-service connections that exist in the workflow under construction.

• The Workflow Diagram provides a graphical display of the current workflow.

**Figure 2:**
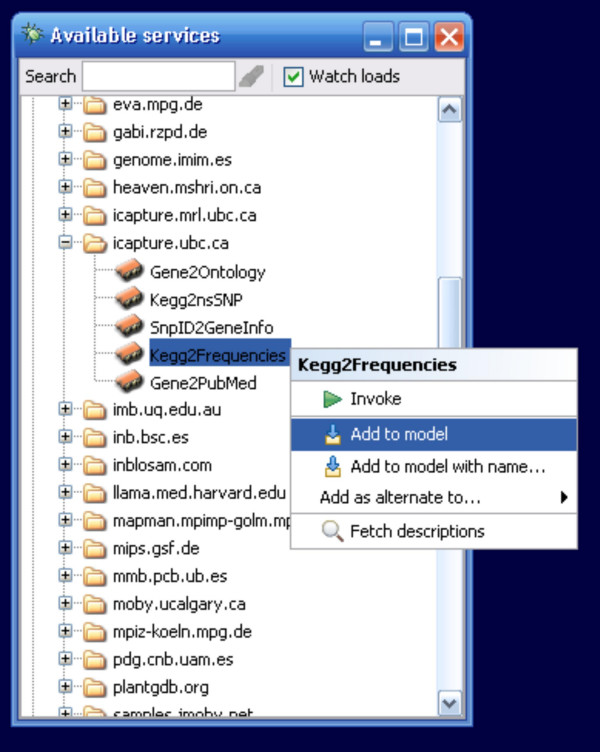
Taverna's Available Services Menu. A BioMoby service "KEGG2Frequencies" has been selected and is about to be added to the workflow model from the pop-up menu. This is how services are traditionally added to a workflow in Taverna.

New widgets are added to the workflow by right-clicking the widget in the Available Services menu and selecting the "add to model" menu option (Figure 2).

**Figure 3:**
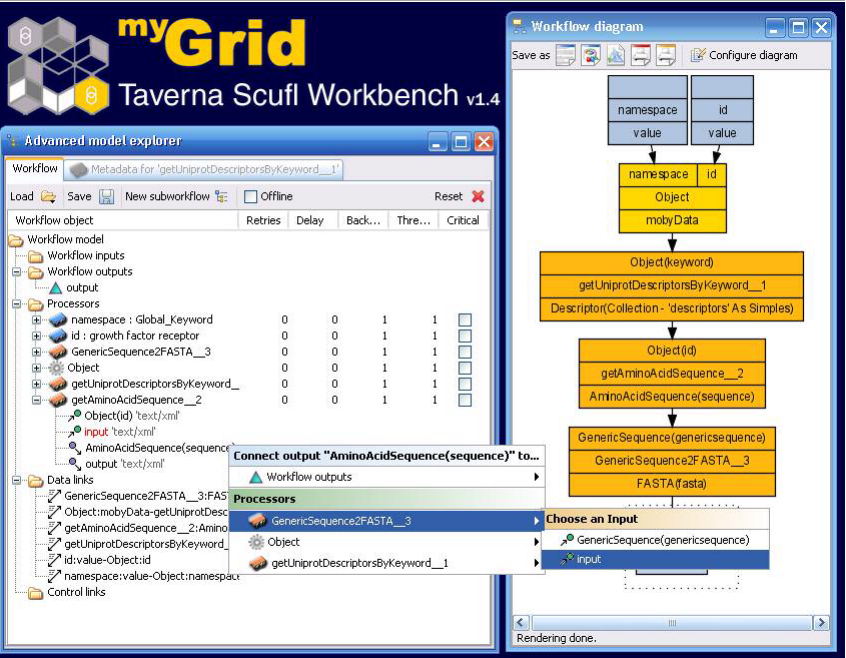
Connecting Services using the Advanced Model Explorer. The output port from the service getAminoAcidSequence is about to be connected to the input port of the GenericSequence2FASTA service. This is accomplished by selecting the output port of the preceding service, right-clicking, and selecting the desired output port from the cascade of pop-up menus.

The inputs and outputs for a widget are called "ports", and the available ports for any given widget can be visualized in the Workflow Diagram, or can be accessed through expanding the Widget tree in the Advanced Model Explorer (Figure 3). Connecting the output of one widget to the input of another is achieved by right-clicking on the output port in the Advanced Model Explorer, and selecting the desired input port from the resulting pop-up menu (Figure 3).

### Taverna BioMoby extensions

A complete, annotated and illustrated example of building a biologically meaningful workflow is available online [18]. Here, in the interest of space and clarity, we will focus only on the individual functionalities that have been added to Taverna by this plug-in, rather than how they might fit together in the context of any given workflow construction exercise. The BioMoby-specific extensions to Taverna include the following:

### Addition of Moby Object constructors/deconstructors

In earlier releases of Taverna, it was possible to construct a base BioMoby data Object consisting of a database namespace and an ID number. Thus all BioMoby workflows in Taverna began with a database identifier. The lack of support for other BioMoby data-types prevented intermediate service outputs from being combined and integrated into more complex objects, and similarly, complex data-types could not be deconstructed to pass sub-components into further downstream services. Often, however, it is desirable to begin a workflow with a more complex data-type, and to construct and deconstruct data-types at arbitrary points along the workflow. This provides access to services, such as the various BLAST services, that consume sequence data with or without an associated identifier.

**Figure 4:**
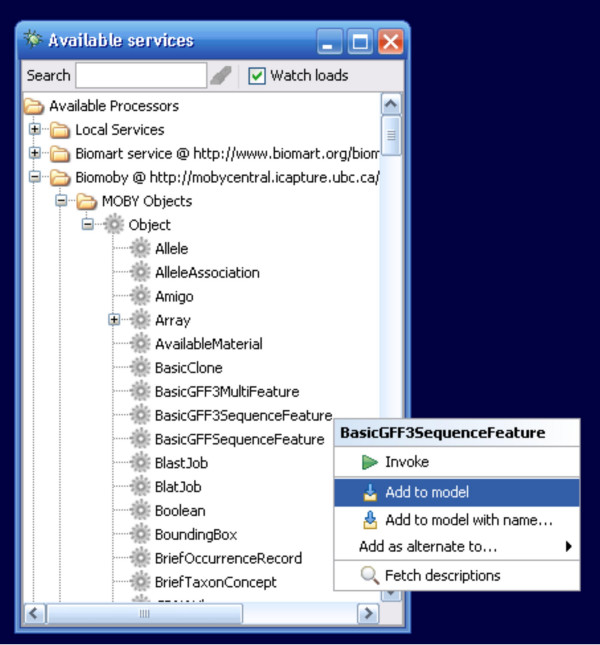
The BioMoby Object Ontology as widgets within the Available Services Menu. The BasicGFF3SequenceFeature widget is about to be added to the workflow.

These limitations have been overcome in the BioMoby plug-in by including the BioMoby Object Ontology as a widget-set in the list of Available Services. These Objects can be added at arbitrary points along the workflow from the usual right-click context menu (Figure 4). Each time Taverna is started the MOBY Central API is used to retrieve a file from MOBY Central describing the full Object Ontology in Resource Description Framework (RDF) format and this is used to assemble the Object widget set, ensuring that it is always up-to-date with the constantly changing BioMoby Object Ontology. Each widget presents all input ports necessary to fill-in the raw data content of that object, as well as an output port from which the resulting BioMoby XML Object construct will be extracted.

**Figure 5:**
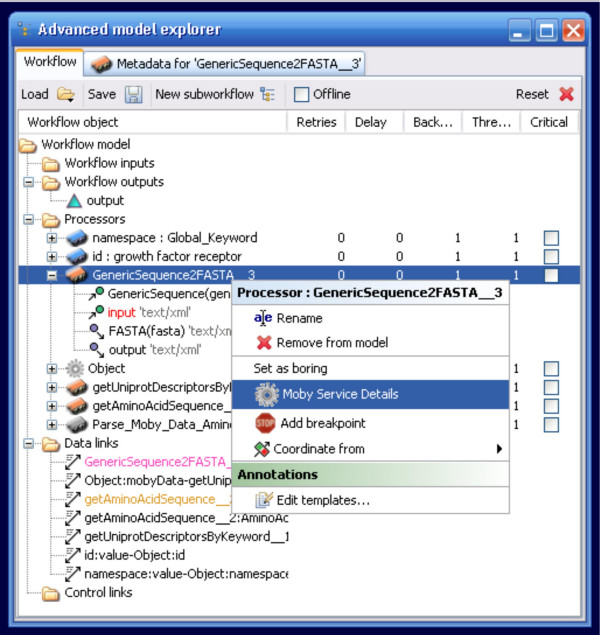
The Moby Service Details option in the Advanced Model Explorer. Unlike non-BioMoby services, the ability to retrieve more advanced details about BioMoby services is made available by the BioMoby plugin as a right-click menu option.

**Figure 6:**
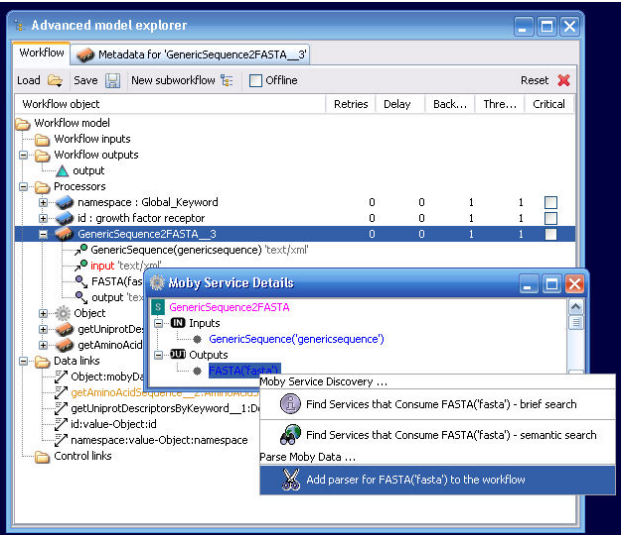
Adding a BioMoby output parser. The output data from any given service can be fed directly into an appropriate XML parser simply by selecting the output data-type from the Moby Service Details window and choosing the "Add a parser" menu option.

Similarly, a set of de-constructor widgets is also now available. Rather than adding these from the Available Services menu, these widgets are accessible from the Moby Service Details context menu, which is accessed through a right-click on the service widget in the Advanced Model Explorer (Figure 5). The Service Details menu displays the input and output of any given service, and by right-clicking on the output the user is provided the option of adding a parser for that output data-type (Figure 6). Upon addition to the workflow, the parser is auto-connected to the appropriate port on the Service. These widgets are crucial for compatibility between BioMoby and non-BioMoby services, since they provide access to the raw content of BioMoby XML objects. Thus it is possible to extract data-types such as strings, integers, and floats, from BioMoby Objects and pass them into non-BioMoby services that consume raw (non-XML) data

### Secondary parameters

In the BioMoby API, a Secondary Parameter is a piece of data that is used to modify the behaviour of service execution, but is not useful for service discovery and is not modeled as a BioMoby Ontologically defined Object – for example, the e-value cut-off of a Blast service.

**Figure 7:**
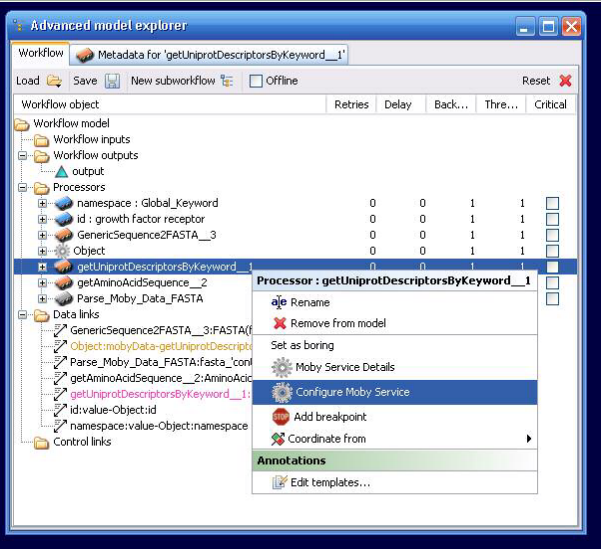
The Configure Moby Service menu option. The BioMoby plugin recognizes when a BioMoby service has additional configuration parameters, and adds an additional menu option to the Advanced Model Explorer's right-click menu.

**Figure 8:**
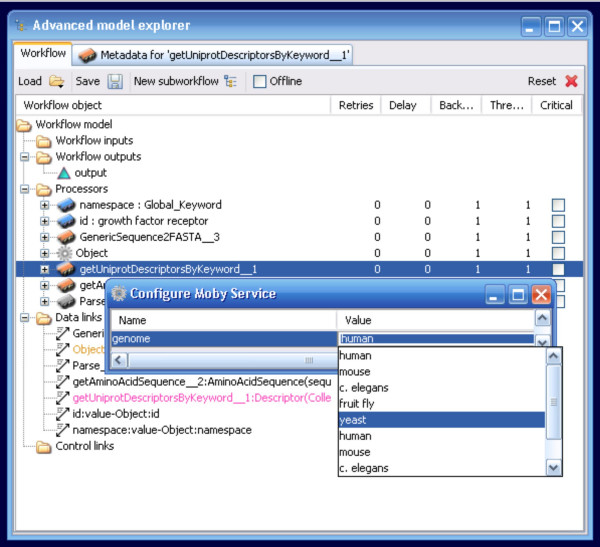
The Configure Moby Service window. All configurable parameters are presented for manual manipulation. In this case, a parameter called "genome" is presented, which is of an "ENUM" data-type, where the different acceptable genomes are listed. The Yeast genome has been selected.

When last queried, about 30% of registered BioMoby services consume secondary parameters. The potential of these services is now fully realized in Taverna by the inclusion of another context-menu option. Right-clicking on a service that requires Secondary Parameter configuration brings up a context-menu that includes the option to "Configure Moby Service" (Figure 7). Selecting this option opens a window in which each configuration parameter is presented with its registered default value selected. Any user modifications of this value are validated against the minimum, maximum, data-type, and/or enumerated values as per the service registration details (Figure 8). This feature was implemented using the Java Secondary Input panel code module from the Moby Dashboard application [19].

### Semantically-aided workflow design

Most, non-Moby Web Services consume strings or numbers as input and produce strings or numbers as output. While these strings generally represent well-recognized bioinformatics data-types, the "intent" of any particular service parameter is opaque to Taverna, and traditional WSDL documents cannot type data more precisely than the limited number of XML schema primitives. BioMoby services, however, consume ontologically-typed data and, as such, services can be discovered that specifically consume these typed data entities. Thus, by making it possible in Taverna to query the BioMoby registry based on particular data-types, workflows could be constructed more intelligently. Moreover this would relieve the burden on the end-user of browsing through the >800 possible BioMoby services in the Available Processors list to identify a service of interest, and ensure that those services presented are guaranteed to be syntactically and semantically compatible in the workflow.

**Figure 9:**
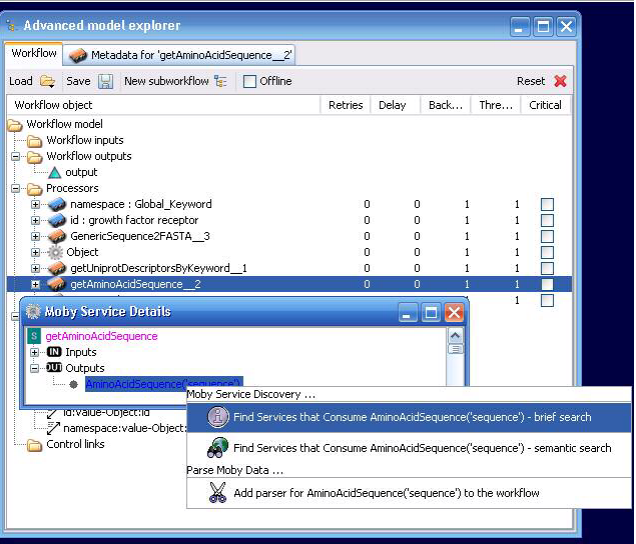
The Moby Service Discovery menu. Unlike any other service in Taverna, the output of a given service can be used to automatically identify all services that are capable of consuming that output, and thus representing the next possible steps in the workflow. There are two ways to achieve the search – with traversal of the Object Ontology (semantic search) or without (brief search). Here, the brief search option has been selected.

**Figure 10:**
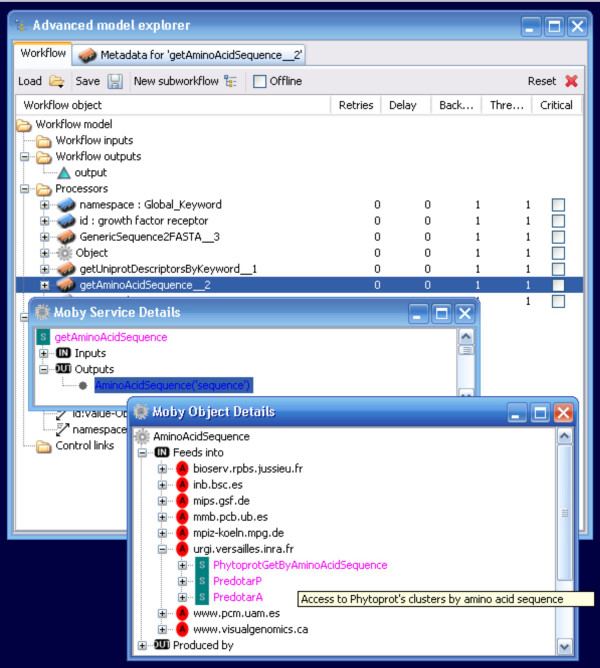
The output from a service discovery. The Object Dertails window displays a list of services capable of consuming the output object from the preceding service. These are organized by service provider (red icons) and all services from a given provider that are capable of consuming that output are presented for selection (purple icons). A mouse-over pop-up help window is visible describing the functionality of the PhytoprotGetByAminoAcidSequence service provided by urgi.versailles.inra.fr.

Service discovery by data-type is supported in the BioMoby plug-in through a right-click context menu in the Moby Service Details window. Two search options are available – search for services that consume the exact data-type (brief search), or search for services that consume the data-type or one of its ontological parents (semantic search) (Figure 9). Discovered services are presented in an expandable menu, organized by service provider (Figure 10), and can be added to the workflow simply by selecting the desired service. Mouse-over pop-up windows provide textual descriptions of what operations each service does. Unlike the operation of adding a service from the Available Processors list, the BioMoby plugin ensures that the connection between the newly added service and the service that feeds into it is created automatically. Thus the creation of BioMoby workflows is not only guided, but is simplified and more rapid than that of non-BioMoby workflows.

### Enhanced support for Simple versus collection inputs and outputs

In BioMoby there are two ways to organize data objects when passing them to a service. Objects that should be processed individually are passed as "Simples", while objects that should be processed as a unit are passed as "Collections" (this is distinct from batch processing, where multiple inputs are passed in the same message, but are treated individually by the service). A collection contains one or more simples of the same data-type.

By default, the BioMoby plug-in to Taverna attempts to make sensible decisions on how to organize data when passing it between services that expect different packages. If an upstream service has just output a Collection (e.g. a Collection of DNA Sequences), and the downstream service it feeds into expects Simples (e.g. a Blast service, that consumes only one DNA Sequence at a time) the decomposition of the Collection into a series of Simples, and the iteration over these Simple inputs to the downstream service is automated. Conversely, if an upstream service is outputting Simples (e.g. a database Sequence retrieval by ID number) and the downstream service expects a Collection (e.g. a ClustalW multiple alignment service), the entirety of the upstream output is "wrapped" in a Collection and passed to the downstream service as a unit.

## Discussion

Taverna is among the most popular Web Service workflow design tools, and is the *de facto *client application for much of the BioMoby user community. Until recently, however, Taverna's support for the most powerful features of the BioMoby interoperability system has been limited. With the BioMoby Taverna plug-in, end-users now have the ability to:

• Initiate workflows using any BioMoby Object of their choice

• Compose and decompose BioMoby Objects to comply with a wider range of Service interfaces

• Parse data out of BioMoby's XML message structure such that it can be used as input to non-BioMoby services

• Assemble raw output data from non-BioMoby services into BioMoby's XML structure

• Access the MOBY Central Web Service registry query system to achieve guidance during workflow construction, thus eliminating the need for the user to have prior knowledge of a particular service interface

• Configure service parameters with guidance and sanity-checking of user-input

• Construct workflows that automatically make correct decisions on how to package data as it passes between the various services.

Certain features that are still not available include the ability to limit semantic searching by the type of service operation, by output data-type, or by keyword; however the myGrid project is currently designing a service query interface, Feta [20], which includes this functionality and will be compatible with both traditional and BioMoby Web Services. As such, these additional features will be available in the near future.

## Conclusion

The majority of functionality exposed by the MOBY Central API is now available via the Taverna graphical interface. When designing a workflow, the user-experience is simplified through a set of guided context-menu choices which ensure that only appropriate services are presented for selection, that selected services are appropriately connected into the workflow, and enable the user to easily move into and out of the BioMoby interoperability framework.

We believe that Taverna is now the most fully-featured client for BioMoby Web Services, and that these enhancements make the Taverna environment more accessible to bench scientists and non-expert bioinformaticians. We are now focusing our development efforts on enhancing the usability and visualization of the output data-sets within the Taverna environment.

## Availability and requirements

**Project Name: **Taverna BioMoby Plugin

**Project Homepage: ** for regular Taverna releases including BioMoby plug-in;  for newest plug-in releases.

**Operating Systems: **Cross-platform

**Programming Language: **Java 1.4 or higher

**Other requirements: **Taverna core code

**License: **GNU Lesser General Public License (LGPL)

**Any restrictions to use by non-academics: **None

## Authors' contributions

MS wrote the original BioMoby Taverna plug-in, with support for base Objects and basic parsing to enable interoperability between BioMoby and non-BioMoby Web Services. MDW created the BioMoby API and designed the extended BioMoby plug-in functionality described here. EK coded this extended plug-in. This manuscript was written by MDW. All authors read and approved the final manuscript.
